# Effect of Different Instrumentation Techniques on Vertical Root Fracture Resistance of Endodontically Treated Teeth

**Published:** 2015-03

**Authors:** Saeid Tavanafar, Azadeh Karimpour, Hamideh Karimpour, Abdulrahman Mohammed Saleh, Musab Hamed Saeed

**Affiliations:** 1 Dept. of Restorative Dentistry, Ajman University of Science and Technology, Ajman, United Arab Emirates;

**Keywords:** Instrumentation, NiTi Instruments, Reciprocation, Root Fracture

## Abstract

**Statement of the Problem:**

Vertical root fractures are catastrophic events that often result in tooth extraction. Many contributing factor are associated with increasing incidence of vertical root fracture. Root canal preparation is one of the predisposing factors which can increase the root susceptibility to vertical fracture.

**Purpose:**

The aim of this study was to compare the effects of three different instrumentation techniques on vertical root fracture resistance of endodontically treated teeth.

**Materials and Methods:**

In this study, 120 freshly extracted mandibular premolar teeth of similar dimensions were decoronated and randomly divided into control (n=30), nickel-titanium hand K-file (HF, n=30), BioRaCe rotary file (BR, n=30), and WaveOne reciprocating single-file (WO, n=30) groups. After cleaning and shaping the root canals, AH26 was used as canal sealer, and obturation was completed using the continuous wave technique. The root canals were embedded vertically in standardised autopolymerising acrylic resin blocks, and subjected to a vertical load to cause vertical root fracture. The forces required to induce fractures were measured using a universal testing machine. ANOVA and Tukey’s post-hoc test were used to analyse the data.

**Results:**

All experimental groups showed statistically significant reductions in fracture resistance as compared with the control group. There was a statistically significant difference between the HF and BR groups. The WO group did not differ significantly from the HF group or the BR group.

**Conclusion:**

All three instrumentation techniques caused weakening of the structure of the roots, and rendered them susceptible to fracture under lesser load than unprepared roots. The fracture resistance of roots prepared with the single-file reciprocating technique was similar to that of those prepared with NiTi hand and rotary instrumentation techniques.

## Introduction

One of the reasons for extraction of endodontically treated teeth is vertical root fracture. Vertical root fracture (VRF) is a longitudinally oriented fracture, extending from the root canal to periodontium. [1] The prevalence reported for VRFs in endodontically treated teeth varies broadly, mostly because of difficulties in its diagnosis.[2-3] The prognosis of root-filled tooth with VRF is very poor and almost always results in extraction of the tooth or resection of the affected root.[4] Some factors such as root canal treatment have been criticised to cause VRF because of its potential to weaken the tooth structure and predispose the tooth to fracture.[5] In a study by Adorno* et al.* (2013), crack initiation was significantly related to preparation, while root canal filling techniques were significantly associated with the propagation of these cracks. Rotary instrumentation has been associated with more cracks compared with hand instrumentation.[6-8] These cracks can gradually degenerate into VRFs. Lam *et al. *(2005) found no increase in fracture susceptibility when comparing the rotary and hand instrumentation.[9]

Advances in nickel-titanium (NiTi) rotary instruments have led to the introduction of canal instrumentation systems with different file designs, metallurgical alloys, and rotational motions. Despite having several advantages compared with the traditional hand instruments, these files are associated with high stress generation within the root canals.[6, 8, 10] Different NiTi instrument designs are associated with different levels of stress and resistance of roots to fractures.[11-12] The single-file reciprocating WaveOne (Dentsply-Maillefer; Ballaigues, Switzerland), an improvement in gradual shaping with multiple instruments, seems to be an attractive option even for novice operators.[13] It is also claimed to be cost-effective and less time-consuming, due to the reduced number of files used compared with the multi-instrument rotary canal preparation techniques.[14] WaveOne is a single-file reciprocating system featuring variable design along with its length, reciprocating motion, and the unique NiTi alloy called ‘M-Wire’. The file cuts counter clockwise (CCW), and its angle of rotation is five times greater in the CCW direction than in the clockwise (CW) direction, which is designed to enhance the resistance of the file to fracture. CW movement of the file disengages the instrument from dentin, relieves the stress as it progresses into the canal, and thereby decreases the chance of taper lock.[15] A study by Burklein* et al.* (2013) compared root canal preparation performed with single-file reciprocating systems with that performed with sequential full rotational files. They showed that defects occurred independently of the instrumentation technique, but reciprocating instruments created more cracks in the apical third of canals.[16] In another study, Ashwinkumar *et al.* (2013) observed more micro-cracks associated with the rotary ProTaper Universal file (Dentsply-Maillefer) than with reciprocating WaveOne or ProTaper hand files, and no micro-cracks with NiTi hand K-files.[7]

This study aimed to compare the differences in fracture resistance of the roots prepared with NiTi hand K-file (HF, Dentsply-Maillefer; Ballaigues, Switzerland), BioRaCe rotary file (BR, FKG Dentaire; La-Chaux-de-Fonds, Switzerland), and large WaveOne reciprocating single-file (WO, Dentsply-Maillefer), in addition to unprepared root canals as control group. Complete canal preparation with a single-file instrument might be assumed to generate more stresses, since only a single file performs the entire enlargement of the canal, which can increase the incidence of dentinal defects, and reduce resistance to VRF. The null hypothesis tested was that there would be no difference between the four groups. 

## Materials and Methods

*Tooth selection*

Prior to conducting the study, the research protocol was approved by the Institutional Ethical Committee of Ajman University of Science and Technology, College of Dentistry (Ref. No. RD-14 (10.03.2013)). Teeth with curved roots, open apices, resorption, or previous root canal treatment were excluded from the collection of human mandibular premolar teeth, which were all extracted in 6 month period for reasons other than for use in this study. The teeth were randomly distributed into four groups (n=30), and cleaned by using an ultrasonic scaler (PerioScan; Bensheim, Germany) and scalpel. Root lengths were standardised to 13 mm, and mean faciolingual and mesiodistal values at this length were obtained. Teeth with more than 20% deviation were replaced with another tooth meeting the previously mentioned criteria. Crowns were sectioned at this length, perpendicular to the long axis of the tooth, with a high-speed fissure diamond bur (Dentsply-Maillefer) using an air turbine (Pana-air; NSK, Japan) at 300,000 rpm under water coolant spray. Presence of a single patent canal was checked with an ISO size 10 K-file (Dentsply-Maillefer) and proximal radiographs. They were then observed under a stereomicroscope with ×30 magnification (BX50; Olympus, Tokyo, Japan), and roots with any fractures or caries were replaced. The 120 teeth meeting the inclusion criteria were kept moist in normal saline throughout the experimental procedure in order to prevent dehydration. 

*Instrumentation*

Tooth working length (WL) was determined by subtracting 1 mm from the length at which a size 10 K-file became visible at the root apex. The reference point was the flat coronal surface of each root. An operator, experienced in rotary and reciprocating systems, prepared all the root canals. Root canals in each group were prepared as follows:

*Control group*

Root canals were irrigated with 1% sodium hypochlorite solution without instrumentation or obturation.

*Step back technique using NiTi hand K-files (HF)*

ISO 0.02 taper NiTi hand K-files (Dentsply-Maillefer) were used in the ISO size sequence 15, 20, 25, 30, 35 and size 40 as the master apical file (MAF) to size 60 as the last file used, with 1 mm incremental reduction from the WL determined by the step back technique. Instruments were regularly cleaned, and root canals were irrigated copiously with 1% sodium hypochlorite solution, followed by recapitulation with the MAF at WL after each step back. Each set of files was used to prepare four root canals.

*BioRaCe rotary NiTi files (BR)*

In accordance with the manufacturer’s recommendations, root canals were instrumented with the crown-down technique using an X-Smart Plus 6:1 Contra Angle (Dentsply-Maillefer) running at 500 rpm with a torque of 1 N.cm. BR0 (25/.08) prepared approximately 4–6 mm of the coronal part of the canals after a hand K-file 15 comfortably reached the WL. Then, BR1 (15/.05), BR2 (25/.04) and BR3 (25/.06) were sequentially used to reach the WL, and final apical preparation was completed with BR4 (35/.04) and BR5 (40/.04) instruments. The blades were cleaned after every four gentle strokes. Root canals were irrigated copiously with 1% sodium hypochlorite solution after each withdrawal of the files, and before changing NiTi instruments. Four canals were prepared with each set of instruments.

*WaveOne single-file reciprocating technique (WO)*

Wave One large files (tip size ISO 40, apical taper of 8%) were used with a WaveOne dedicated motor (Dentsply, Maillefer) set to the configuration recommended by the manufacturer. Roots were instrumented through a progressive up and down motion with little force in no more than three to four times. The files were removed after every three to four pecks, wiped clean, and root canals were then irrigated copiously with 1% sodium hypochlorite solution. Four canals were prepared with each instrument.

*Obturation*

Root canals in all groups were dried with paper points and obturated using an Element Obturation Unit (Sybron-Endo; Sybron Dental Specialties Inc., Glendora, CA, USA) with a master gutta-percha cone size of 40, and a continuous wave of warm gutta-percha (Elements gutta-percha cartridge; 23 gauges, Sybron-Endo, USA). AH26 (Dentsply DeTrey; Germany) was used as root canal sealer. Post-operative radiographs confirmed the quality of the obturation. Coronal and apical parts of the roots were covered with double layer of nail polish and stored in normal saline for 2 weeks, allowing the sealer to set and also preventing root dehydration.

*Mounting the roots and measuring fracture resistance*

All the roots were mounted vertically in standardised cylindrical autopolymerising acrylic with diameter of 13mm and length of 14mm (Meliodent; Bayer UK Limited, Newbury, UK). The roots were positioned at the centre of the acrylic resin, and covered with a very thin layer of wax (0.2–0.3 mm) such that 12 mm of the root was retained inside the mounting. After setting of the acrylic resin, the roots were removed and any remaining wax was washed out. A thin layer of polyvinyl siloxane (Speedex; Light Body, Coltene, Switzerland) was applied to the cavity of the root inside the acrylic resin, and the roots were returned to the same position, thus simulating periodontal ligament.

The centre of the coronal surface of the root canal filling material was continuously loaded by perpendicular external static force applied with a stainless steel parallel rod (0.7-mm diameter flat end). Obturation material acted as a medium to distribute the force. A universal testing machine (Instron Corp.; Canton, MA, USA) was used, operating at a cross-head speed of 1.0 mm/min. The rod was inserted into the root canal to contact gutta-percha, and distribute the load to the canal walls. Tests started with the machine gradually applying force to the canal through the gutta-percha, without touching the walls. In control group, the load was directed to the canal lumen. The machine was stopped immediately after detecting fracture or by sudden reduction in load. The load at fracture time was recorded in Newton. The maximum load during each test was defined as the fracture load. After each fracture test, the roots were dyed with 2% methylene blue dye solution and viewed under magnification, to confirm the fracture and determine the pattern of the fracture lines to be buccolingually, mesiodistal, or compound.

*Statistical analysis* **

Data were analysed using the Kolmogorov-Smirnov normality test and ANOVA. Once a significant difference in score was found (*p*< 0.05), Tukey’s post-hoc test was used to determine significant differences in average scores between specific groups. All statistical analyses were performed at a 95% level of confidence. IBM SPSS (ver. 21.0; SPSS Inc., Chicago, IL, USA) was used to conduct all analyses.

## Results

No instrument fractured during instrumentation. Table 1 shows the mean load at fracture in each group.

**Table 1:** Mean±SD of loads at fracture in Newton (N)

**Instrument**	**Mean±SD**	**Minimum**	**Maximum**	**Range**
Control	303 ± 60^a^****	202	442	240
NiTi hand K-file	264 ± 54^b^	182	364	182
BioRaCe	198 ± 43^c^	124	295	171
WaveOne	234 ± 57^b,c^	155	335	180

Values with the same superscript letters did not differ significantly from each other at *p*< 0.05.

**Figure 1:**
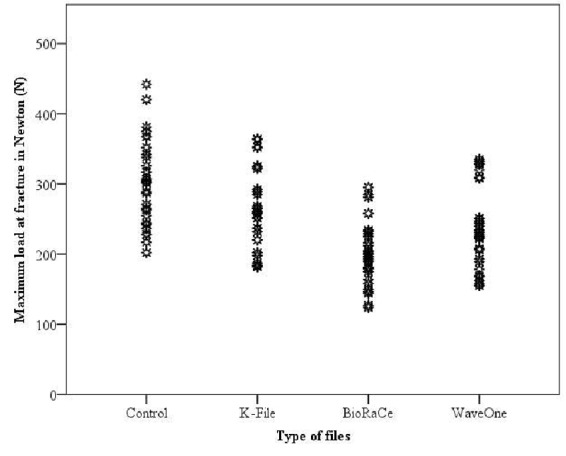
Assessment of loads at fracture following biomechanical preparation, obturation, and loading. n=30 for each group (*p*< 0.05)

WO did not differ significantly from HK (*p*= 0.15), or BR (*p*= 0.06), but there was a significant difference between HK and BR (*p*< 0.0001). The control group differed significantly from all of the experimental groups (*p*< 0.05), and it had a wider range of load at fracture than the experimental groups (Figure 1). Being seen in 96 roots (80.0%), buccolingual direction of fracture was the most common pattern of fracture, followed by the mesiodistal direction, in 21 roots (17.5%). Compound fracture was only seen in three roots (2.5%).

## Discussion

The catastrophic event of VRF leads to tooth extraction or root resection. Instrumentation may contribute to VRF by inducing stress or through excessive dentinal removal. NiTi K-file, BioRaCe, and WaveOne were chosen because each represents different instrumentation techniques featuring different cross-sectional geometry, taper, flute form, type of manufactured alloy, number of instruments used, and rotational motion that can influence tooth resistance to VRF. In this study, no difference in fracture resistance was evident between roots prepared with single-file WaveOne when compared with either hand NiTi K-files or the rotary NiTi BioRaCe instrument (*p*> 0.05). The control group was more resistant to fracture than all other groups (*p*< 0.05), suggestive of roots becoming more susceptible to fracture regardless of instrumentation technique; which was consistent with the findings of previous studies.[10, 17]

File design can result in dentinal defects and reduce the fracture resistance of roots.[11] Stiffer files generate higher stress concentration. Stiffness is related to size, taper, cross-section, method of manufacturing, and the material out of which the instrument is made.[18] M-Wire is a more flexible type of conventional NiTi from which WaveOne instruments are made.[19] Concerning the designs of the files used in this study, WaveOne files feature a modified convex triangle with radial lands from D1 to D8, and a convex triangle from D9 to D16, while, the BioRaCe has reverse-acting cutting edges with a simple triangular cross section. Evidently, instruments with a triangular cross-section have more even stress distributions along their length, and lower stress concentrations, than the instruments with rectangular cross-sectional designs, which can create higher stress differentials during simulated canal shaping.[18]

Canal diameter can also affect the root resistance to vertical fracture.[12] It is not surprising that removing more dentin reduces the fracture resistance of roots.[20-21] Excessive dentin removal would unnecessarily weaken and compromise the structural integrity of roots; thus it should be avoided. Additionally, more craze lines are found in areas with more root structure removed,[20] and the larger the diameter of the canal, the less the resistance to fracture.[22] In this study, the apical diameters of all experimental groups were similar and there was no significant difference despite WaveOne having a greater taper than BioRaCe (0.04 for the final finishing files) and hand NiTi K-files (0.02).[19] This could be because of the difference in their rotational motion. Conventional full rotation can cause significant micro-crack formation, via the constant torque applied by NiTi rotary instruments on the root canal wall. These stresses can create defects and damage the dentinal walls which can progress to VRFs.[7-8] Reciprocating motion works by alternately disengaging dentin, releasing the stress on the file and dentinal walls, and this could be a contributing factor to greater root resistance to fracture, even where the greater taper of reciprocating files results in reduced remaining dentinal thickness. On the other hand, Sathorn* et al.* (2005) studied the effects of intrinsic factors of roots on fracture susceptibility and pattern, and found that dentinal removal is not the only factor associated with reduced fracture resistance, and does not always result in increased fracture susceptibility.[21] Rather, root fracture results from interaction between multiple factors with intrinsic aspects of the canal playing an important role.

The predominant buccolingual fracture pattern observed, followed by proximal then compound patterns, was consistent with previous researches.[9, 23] Sathorn *et al.* (2005) observed more mesiodistal patterns of fracture in mandibular incisors, and hypothesised that NiTi instrumentation might have changed the pattern.[22]

The wide variation in load at fracture observed in the control group (Figure 1) could be the result of uneven stress distribution in this group. Less variation in other groups could be the result of dissipating stresses and preventing them from being concentrated on a particular point, which is the characteristic of smooth preparations.[9, 22] While canal morphology and the external shape of roots can significantly affect the fracture resistance of a root,[21] clinicians can limit VRF occurrence through identifying susceptible teeth by their intrinsic factors, and adopting conservative and valid clinical principles when treating these teeth.

Human mandibular premolars have been used because of their similarity in shape.[24] They are also usually extracted for orthodontic purposes, thus they could be collected easier. The roots in this study were subjected to static vertical force until fracture similar to previous studies.[9, 22-23] however; in a clinical situation, roots should withstand different forces during and after root canal treatment. In this study, it was also assumed that gutta-percha equally and uniformly distributes the vertical load around the canal wall. In practice, this could not be usually achieved. The spreaders might touch the wall in lateral condensation techniques, and a point load would be resulted. Also, root dentine sclerosis in relation to the age and race of the patients, which can affect the strength of root[25] were not recognized in this study. Despite these limitations and assumptions, characterising the weakening of roots during root canal treatments in controlled laboratory conditions may assist clinicians in adapting to different NiTi instrumentation techniques. This *in vitro* study can give an indication of root susceptibility to VRF when subjected to forces encountered in clinical situation such as obturation, post placement, and subsequent clinical function. Considering root fracture in a multi-factorial context, the WaveOne reciprocating single-file system was comparable to NiTi hand K-files and sequential BioRaCe rotary files with regard to root fracture resistance. Further research should evaluate their shaping abilities in comparison with different instrument systems.

## Conclusion

Root canal treatment weakens roots, and in this study, instrumentation with the single-file reciprocating technique was associated with resistance to fracture comparable with the roots prepared with NiTi hand or rotary instruments. 
